# The Public Health—Child Welfare in the West Riding

**Published:** 1923-02

**Authors:** 


### THE PUBLIC HEALTH.

### INTERVIEWS WITH LOCAL AUTHORITIES.

### CHILD WELFARE IN THE WEST RIDING.

YORKSHIRE, as everyone knows, is the county of broad acres. It is not so many years ago, as years of human history go, that a visitor to the West Riding, inquiring of a shepherd how many sheep there were to an acre, was rebuked with the statement that he was putting his question the wrong way round and that the number of acres to a sheep should more properly have formed the subject of his inquiry. The Public Health and Housing Committee of the West Riding County Council have to-day a problem and a responsibility which embrace rural conditions certainly, but it is mainly a problem and a responsibility of industrial and urban conditions created by the tremendous growth of the textile, mining and other large industries for which this vast area is famous. Notwithstanding the number of large County Boroughs which have from time to time assumed their own responsibilities, some idea of the character of the West Riding area of 1,650,000 acres may be gauged from the fact that it comprises 11 boroughs and 114 urban districts, as compared with only 28 rural districts. The population of the West Riding as a whole has increased sevenfold in the last century. It is safe to say that the extent and cost of the health activities of the county have increased even more than this within the period during which Dr. J. R. Kaye has rendered some 30 years' faithful service as the County's Medical Officer. Notwithstanding the heavy task of maintaining the existing health activities, the developments which the future has in store for the County's Health Committee might well stagger health workers even so energetic and enthusiastic as the Chairman of the Committee, Alderman Ben Turner, M.P., his colleague, Canon Phipps, the Deputy Chairman, and so experienced a Medical Officer as Dr. Kaye. These three gentlemen, who received us on our visit to Wakefield, dealt very fully and courteously with our inquiries and gave us a very interesting expression of their views and an account of their work.

#### The County Council and the Ministry.

We were not surprised to learn that the sturdy and independent Yorkshireman finds irksome, however gently it is exercised, the amount of detailed control of the Ministry of Health which appears to be the inevitable accompaniment of Exchequer assistance for health services. It often spells a certain amount of delay, and your Yorkshireman wants to shake it off and get on with the job. The West Riding of Yorkshire feels that it can make quite good progress without too much interference from Whitehall, though it is recognised that some measure of interference is unavoidable if Exchequer assistance in aid of health work is to continue to be available. The Health Committee think that such assistance should continue, to encourage local activity and to render the burden more remote; that it does not remove the burden is, of course, frankly recognised, because the Yorkshire taxpayer will clearly have to pay his share. Also the collective wisdom and experience of the country as a whole—and the West Riding could contribute no mean share—should be distilled in Whitehall, and generally available. "We do not at all object to being educated," said Alderman Ben Turner, "and equally we expect Whitehall to have the general power of approval of schemes as a whole and especially new schemes; that is, of course, where Exchequer help is given. But once the scheme is approved, and subject to a broad survey of the results, we expect to be left to work out the items and have more or less a free hand in the detailed spending of the money."

#### A Domesday Book.

Dr. Kaye has been engaged for many years past in the preparation of what he calls his Domesday Book. For every Union in the county there is being prepared, and printed in permanent form, an exhaustive survey of the Union area, its population, rateable value, vital statistics over a period of years, details of analysis of samples of drinking water, particulars of all sewerage and drainage arrangements, statistics in great detail, of dwellings and tenements, school accommodation and other valuable information. So comprehensive is the survey that such details are given, with regard to cowsheds examined, as cubic feet per animal, measurements of windows, character of flooring, &c. The defects throughout each area are all carefully recorded. In a copy of the report of the survey of one of the urban areas, taken at random, we observe: No efficient hospital, no disinfecting apparatus, no ambulance. Public scavenging? No. Baths and washhouses. No. There is also in the same report a reference to the suspicious quality of the water in certain districts, in some others there is recorded a scarcity of water, and also that it had to be carried an unreasonable distance in many cases. These things, it may be observed, occur in part of a Union area, mountainous, with a configuration and rainfall rendering it suitable for, and, in fact, largely utilised as gathering grounds for public water supplies.

#### Co-ordination of Health Services Needed.

Alderman Turner and his colleagues constantly feel the need for such a widening of their powers as will enable them more effectively to get things done. It is too often their experience that after grave defects have been revealed and pressure brought to bear upon the sanitary authorities to put the matters right, they remain unremedied. Apart from this, however, they feel that there are too many kinds of authorities all concerning themselves with the same subject. Admittedly there is some overlapping, but Mr. Turner sagely remarked that the greatest evil of divided responsibility was not overlapping, but what he called underlapping, or a failure to get things done. The need for co-ordination is everywhere evident in a county such as the West Riding, and it is hopeless to expect that substantial progress shall be made in the attack on the common enemy—disease, until all the forms of health services are brought into one organised plan, under one general supervision. The sanitary services, the Poor Law health services, the medical treatment of insured persons, the various special services directly administered by the county—such as tuberculosis, maternity and child welfare—all having one common aim, that of the constant betterment of the health of the people, need to be properly related to one another and subject to a common guidance. But, coupled with this central guidance and co-ordination, Mr. Turner was careful to insist that there must be a wide and a wise devolution, so that local interest and local spirit is fostered and encouraged everywhere.

### The Vital Statistics.

Meanwhile, in the matters for which they have a direct responsibility for a population of a million and a half of people, the Health Committee have no inconsiderable task. It may be convenient here to give a short summary of the vital statistics, as follows :—

| | Average for 10 years. 1911-20. | 1920. | 1921. |
|---|---|---|---|
| Birth Rate (Administrative County) .. .. .. | 22·8 | 25·2 | 23·3 |
| Per 1,000 estimated population. | | | |
| Death Rate .. .. .. | 14·3 | 12·6 | 12·6 |
| Zymotic Death Rate .. | 1·23 | 0·95 | 0·78 |
| Phthisis Death Rate .. | 0·83 | 0·71 | 0·74 |
| Respiratory Death Rate .. | 2·57 | 2·27 | 2·20 |
| Per 1,000 estimated civil population. | | | |
| Infantile Mortality (Number of deaths under one year per 1,000 Births) .. .. .. | 109 | 92 | 97 |

The figures of infantile mortality compare unfavourably with those for England and Wales. Bronchitis and pneumonia especially are responsible for a heavy mortality, and the high average rate is generally found to be due to the loading of the serious figures of the colliery districts. From an analysis of the figures it appears that rates varying from 120 to so grave a figure as 179 are found to exist almost entirely in colliery districts, or those having manufactures closely dependent for their activity on the coal industry.

### Child Welfare.

Where the infant mortality is heavy the root causes are to be found, as indicated, in environment, with which the maternity nurse and the child welfare worker are largely powerless to deal. But the growth of child welfare work in recent years is evidence that authorities feel that they cannot afford to wait while housing conditions get better. There has until recent years been a preponderance of untrained over trained midwives in the West Riding, but a steady increase in the latter is now manifesting itself, the number of trained and untrained midwives in 1921 being respectively 326 and 255. In connection with the care of babies, two special features in the administration call for special mention—the stress which is laid on the importance of the breast-feeding of infants ; and the doubt which the Medical Officer throws on the value of baby competitions. As to the first, Dr. Kaye urges that every child welfare centre should be the means of inculcating and spreading correct ideas on infant feeding, of which the first in importance is the immense superiority of breast feeding over all other methods of feeding. It should be the aim, he says, of every Medical Officer to encourage every mother to persevere in the attempt to nurse her baby, even where it seems at first most hopeless. It has been demonstrated that even as late as six weeks after the birth of her child a mother may become able to nurse it herself, and that nothing short of the actual destruction of breast tissue can destroy the power of lactation in a mother. Yet notwithstanding this there are often many difficulties in establishing and maintaining a sufficient supply of breast milk. Many mothers are daunted and discouraged by these difficulties and too readily give up the attempt. The success of a child welfare centre, it is thought, should be measured, not by swollen attendances or gigantic sales of dried milk, but by the percentage of mothers who are nursing their babies. As the centres are used for the distribution of dried milk, and the mothers see considerable activity in this department, there is some danger of their concluding that dried milk is the method of feeding for infants which has official approval.

### Competitions for Fathers.

With regard to competitions designed to encourage the care and development of the baby, our attention was specially drawn to the work of the Knaresborough Child Welfare Committee. This Committee, instead of holding a baby competition, organised a competition with the following items : A written examination on the subjects of the lectures given ; bread baking ; laundry work ; economic garments—that is, garments made from the serviceable remains of adult clothes ; and, in order to suggest to fathers that paternal resposibility for children is not so limited as sometimes their comparative indifference suggests, a competition for fathers in home-made toys.

### Making Real Homes.

Competition on these lines, creating a healthy rivalry, might, it is thought, supersede " Baby Competitions." Several baby competitions have been immediately followed by a drop in attendance at the centres, because while the award of prizes pleases the winners it offends all the others. It is impossible accurately to assess the merit of the mother by the condition of her child, for the circumstances of the competitors may be widely different. The babies do not all start from " scratch." The mothers are differently handicapped, and the judges do not know the mothers' handicap. A competition for mothers (and fathers) which Dr. Kaye would like would be one for the greatest resourcefulness and ingenuity in converting an unpromising house into a real home, or for such inventions as home-made waterproof awnings for perambulators, so that babies could be out of doors almost the whole of every day.

